# Patterns of Father Involvement and Child Development among Families with Low Income

**DOI:** 10.3390/children8121164

**Published:** 2021-12-09

**Authors:** Susan Yoon, Minjung Kim, Junyeong Yang, Joyce Y. Lee, Anika Latelle, Jingyi Wang, Yiran Zhang, Sarah Schoppe-Sullivan

**Affiliations:** 1 College of Social Work, The Ohio State University, Columbus, OH 43210, USA; lee.10148@osu.edu (J.Y.L.); latelle.1@buckeyemail.osu.edu (A.L.); zhang.7107@buckeyemail.osu.edu (Y.Z.); 2 Quantitative Research, Evaluation and Measurement, College of Education and Human Ecology, The Ohio State University, Columbus, OH 43210, USA; kim.7144@osu.edu (M.K.); yang.5631@buckeyemail.osu.edu (J.Y.); 3 Department of Psychology, College of Arts and Sciences, The Ohio State University, Columbus, OH 43210, USA; wang.12699@buckeyemail.osu.edu (J.W.); schoppe-sullivan.1@osu.edu (S.S.-S.)

**Keywords:** father involvement, child development, socioemotional functioning, behavior problems, cognitive functioning, latent profile analysis

## Abstract

This study examined patterns of father involvement and their relations with social, behavioral, and cognitive development among low-income children < 5 years. Latent class analysis on data from 2650 fathers (Mage = 29.35 years) in the Supporting Healthy Marriages program revealed four father involvement patterns: (1) High positive involvement (48%); (2) engaged but harsh discipline (42%); (3) low cognitive stimulation (8%); and (4) lower involvement (2%). The low cognitive stimulation pattern was associated with greater father- and mother-reported child behavior problems and lower child socioemotional and cognitive functioning. The engaged but harsh discipline pattern was associated with more father-reported child behavior problems. These findings highlight the need for active engagement of fathers in parenting interventions to promote child development.

## 1. Introduction

Father involvement is a key family protective factor that is crucial to children’s healthy development [1,2,3,4]. Numerous studies suggest a link between greater father involvement and child positive health [5,6,7], mental health [8,9,10], socioemotional [10], academic [11,12], and behavioral outcomes [9,13,14]. However, various patterns of father involvement across multiple dimensions of functioning and their unique impacts on healthy child development across the social, behavioral, and cognitive domains remain unclear, especially among economically disadvantaged families. Approximately 17% of children in the United States live in families with low income [15], and children born to parents with low income tend to have poorer developmental outcomes [16,17,18]. However, not all children in families with low income have poor developmental outcomes. Research has suggested that early parent involvement can have both short- and long-term positive effects on child development in families with low income [18]. Further, there is preliminary evidence that the positive impact of father involvement on child academic outcomes is stronger for children in families with low income than those in middle- and upper-income families [19]. It is vital to examine whether such benefits of father involvement on child academic outcomes among children in families with low income extend to other domains of development (e.g., social and behavioral) for this population. In sum, identifying distinctive patterns of father involvement and their contributions to diverse aspects of child development among families with low income is an important focus of inquiry that can inform the development of interventions to promote healthy development in vulnerable children. 

### 1.1. Father Involvement and Child Development

As fathers’ roles expanded in the 1970s to encompass caregiving in addition to breadwinning [20], scholars’ recognition that fathers could make positive contributions to their children’s development increased. Although early research—especially that focused on father involvement in lower-income families—tended to use relatively simple measures of accessibility (e.g., presence vs. absence of father in the home), this focus soon expanded to encompass time fathers spent engaging in play, cognitively stimulating, or caregiving activities with children [21,22]. Overall, greater father involvement in childhood has been associated with healthier child development in cognitive, social, and behavioral domains [4,23]. For example, a study involving children between 3 months to 24 months illustrated a positive contribution of father engagement to higher cognitive functioning [24]. Similarly, a meta-analysis involving 21 studies concluded that father involvement was consistently found to have a small to moderate positive effect on children’s early learning [3]. 

### 1.2. Heterogeneity in Father Involvement and Child Development 

Fathering has long been viewed as a multidimensional construct [22]. In the mid-1980s, Lamb and Pleck proposed a three-dimensional conceptualization of fathering [25]. This model posited that father involvement was primarily composed of paternal engagement, accessibility, and responsibility. While Lamb and Pleck’s model provided one of the first frameworks for understanding the complexity of father involvement, it was far from comprehensive. Scholars subsequently expanded on this model by including dimensions related to communication, father–child closeness, and time spent with the child [26]. 

Pleck later proposed a revision to the original model including five dimensions of father involvement [22]. Pleck asserted that fathering involved three direct or primary activities classified as “(a) positive engagement activities, interaction with the child of the more intensive kind likely to promote development; (b) warmth and responsiveness; and (c) control, particularly monitoring and decision making” [22] (p. 67). Pleck also included two ancillary domains: Indirect care and process responsibility [22]. Indirect care encompasses activities that are conducted for a child but that do not directly involve father–child interactions, such as the purchase of school supplies. Process responsibility refers to a father’s oversight that their child’s core needs (e.g., positive engagement, warmth) are being met. 

Several empirical studies have suggested that different aspects of father involvement may be associated with different dimensions of child development. For example, positive father–child relationships, paternal warmth, and home learning stimulation have been associated with stronger socioemotional development (e.g., social competence, prosocial skills) and cognitive development, whereas paternal harsh parenting has been associated with greater behavior problems, such as childhood aggression [23,27,28,29]. Positive father–child relationships have been linked to reduced internalizing and externalizing problems in children and adolescents. For example, high-quality father involvement, which includes trust, closeness, and understanding, was associated with fewer internalizing and externalizing symptoms in a sample of children at risk for maltreatment [30]. Similarly, a recent study found that fathers’ early involvement was associated with lower levels of children’s internalizing and externalizing problems [23]. 

Few studies have considered the complex interaction of multiple dimensions of father involvement or examined heterogeneous patterns of involvement and their relations with children’s social, behavioral, and cognitive development, although some relevant evidence is emerging [31,32]. For example, Volling et al. used a middle-income sample of 195 two-parent families with 12-month-old infants to examine fathers’ (and mothers’) parenting profiles, with a particular focus on fathers’ engagement in behaviors that excite and stimulate their children and are posited to contribute to their children’s development [32]. Results of latent profile analysis showed that fathers and mothers had similar (a) supportive (i.e., high levels of sensitivity, positive regard, and cognitive stimulation); (b) disengaged (i.e., high levels of detachment); and (c) activation (i.e., moderate levels of sensitivity, positive regard, cognitive stimulation, and intrusiveness) parenting profiles although none of the parenting profiles were related to infants’ attachment security. 

Researchers have also investigated the parenting patterns of fathers (and mothers) from low-income backgrounds, although to the best of our knowledge, the literature seems to be limited to the following two studies. Ryan et al. used an Early Head Start sample of 237 two-parent families with 2-year-old children to examine fathers’ and mothers’ parenting profiles [33]. A person-centered cluster analysis revealed four distinct parenting profiles for both fathers and mothers: (a) Highly supportive (e.g., high levels of sensitivity, positive regard, and cognitive stimulation); (b) negative (e.g., high levels of intrusiveness); (c) detached (e.g., high levels of detachment); and (d) somewhat supportive (e.g., moderate levels of sensitivity, positive regard, cognitive stimulation, and intrusiveness). The researchers further showed that children with a supportive father and supportive mother had the best cognitive functioning compared to all other children. 

More recently, Lee et al. aimed to replicate prior research by using a sample of 672 two-parent families with preschoolers from the Building Strong Families project, a large and racially diverse dataset of families from socioeconomically disadvantaged backgrounds [34]. Results of latent profile analysis yielded three parenting profiles for both fathers and mothers: (a) Supportive (e.g., high levels of sensitivity, positive regard, cognitive stimulation); (b) intrusive (e.g., high levels of intrusiveness); and (c) activation (e.g., moderate levels of sensitivity, positive regard, cognitive stimulation, and intrusiveness). Consistent with Ryan et al., children with a supportive father and supportive mother had the highest language scores compared to all other children [33]. That said, when it came to socioemotional outcomes (e.g., prosocial behaviors, behavior problems, effortful control), children with an activation father and a supportive mother did just as well as those with two supportive parents. Overall, there seems to be consensus across these prior studies about the heterogeneity in father involvement, with multiple parenting profiles emerging, and their differential effects on child development. While these studies provided valuable information, they were limited in that they primarily focused on the quality of involvement and did not consider both the quality *and* quantity of father involvement. 

### 1.3. The Current Study 

Despite emerging evidence suggesting heterogeneity in father involvement, additional research is needed to understand the patterns of father involvement among low-income, racially diverse families and their relations to various dimensions of child development. The current study has several significant contributions. First, we apply a person-centered analytic approach (i.e., latent class analysis)—an effective method also used by prior research in this area that allows for identification of subgroups of individuals based on their particular attributes—to investigate heterogeneous patterns of father involvement, going beyond the traditional variable-centered approach [35]. Second, we combine both quantity and quality of measures of father involvement to better capture various patterns of father involvement. Most past studies of fathering/parenting profile focused on the quality of father involvement, despite empirical evidence suggesting the importance of conjointly considering both the quality and quantity of father involvement [36]. Third, this study is novel and different from prior research in that we examined harsh discipline, an important yet often ignored dimension of involvement, along with other aspects of father involvement. Finally, we use a larger sample of low-income, racially/ethnically diverse families to expand our understanding of the role of father involvement patterns in healthy child development in marginalized and diverse populations.

The current study aimed to discover various patterns of father involvement and their unique relations to social, behavioral, and cognitive development of children in families with low income. Two main research questions guided the study: (1) Are there different patterns of father involvement among families with low income? (2) How do different patterns of father involvement relate to social competence, behavior problems, and verbal ability of children? Building upon Pleck’s conceptual model that highlights multidimensionality of father involvement and prior studies that identified heterogeneous parenting profiles [22,32,34], it was hypothesized that approximately four different patterns of father involvement (e.g., supportive, detached, intrusiveness/negative, activation) would emerge in this study. Informed by prior evidence [33,34], it was further hypothesized that more positive patterns of father involvement (e.g., high warmth and engagement, no harsh discipline) would be associated with higher social competence, fewer internalizing and externalizing behavior problems, and higher verbal ability in children. 

## 2. Methods

### 2.1. Participants and Study Design and Procedure

We conducted a secondary data analysis using data from the Supporting Healthy Marriages (SHM) program, which is a multisite, voluntary marriage education program for low-income couples who had a child under 18 years old or were expecting a child. The SHM project used an experimental study design. A total of 6298 families were recruited and randomly assigned into the intervention or the control group, from February 2007 to December 2009. The program offered group workshops, supplemental activities, and family support services that were designed to strengthen couples’ relationships. Three waves of data were collected: (1) When eligible couples first enrolled in the program and completed the baseline survey (during this time period, researchers also randomly selected one child from each couple as the focal child for follow-up studies); (2) 12 months after enrollment when both survey and observational data were collected from the couples; and (3) 30 months after enrollment when couples completed a series of surveys and a subgroup of focal children participated in direct assessments. At the 12-month and 30-month follow-up survey interviews, the participants were given the option of using the computer-assisted telephone interview (CATI) method or the computer-assisted in-person interview (CAPI) method to respond to the survey questions.

In the current study, we primarily used data from the 12- and 30-month follow-up assessments. The following criteria were adopted to determine the analytic sample: (a) At the 12-month follow-up, focal children were 4 years and 11 months old or younger; (b) at the 12-month follow-up, fathers had contact (e.g., in person, text message, phone call, email) with focal children in the past month; and (c) families did not have missing data on all variables of interest. As a result, 2650 families were included. When eligible couples enrolled in the program, their ages ranged from 21 to 40 years old (*M*_father_ = 29.35, *SD*_father_ = 5.84; *M*_mother_ = 27.40, *SD*_mother_ = 5.26). Amongst the eligible families, 51.79% of the focal children were boys and 48.21% girls. The sample was diverse in terms of race and ethnicity. For fathers, 45.60% identified as White, 19.23% African American, 2.40% Asian, 4.16% American Indian/Alaska Native, 1.47% Pacific Islanders, and 27.14% other races. For mothers, 48.81% identified as White, 14.70% African American, 3.20% Asian, 4.17% American Indian/Alaska Native, 1.58% Pacific Islanders, and 27.54% other races. Moreover, 40.48% of fathers and 40.65% of mothers identified as Hispanic. Most families had low household income, with 38.12% having income below the federal poverty level (FPL), 41.99% between 100 and 200 percent of the FPL, and 19.89% above 200 percent of the FPL. In terms of fathers’ residential status, 97.25% of fathers at the 12-month follow-up study and 91.97% of fathers at the 30-month follow-up study reported that they lived with the focal child at least half of the time. Table 1 further provides the demographic characteristics of study participants.

### 2.2. Measures

#### 2.2.1. Father Involvement

At the 12-month follow-up assessment, fathers reported their involvement with the focal child. Fathers reported frequencies of activities and behaviors they engaged with their child in the past month. The survey included the following domains of father involvement: (1) One item indicating time spent with the child (i.e., “Spend one or more hours a day with the child”); (2) five items indicating engagement in caregiving, play, and cognitively stimulating activities (i.e., “Played inside with games or toys”, “Taken the child for a walk”, “Sung songs or nursery rhymes with the child”, “Read books or told stories to the child”, “Dealt with the children when he/she did something wrong”); (3) three items indicating parental warmth (i.e., “Told the child that you love him/her”, “Praised the child or told him/her that you appreciated something that he/she did”, “Laughed with the child”); and (4) two items indicating harsh discipline (i.e., “Yelled, shouted, screamed at, or threatened the child because you were mad at him/her”, “Hit, spanked, grabbed, or used physical punishment with the focal child”). That is, a total of 11 items pertaining to different domains of father involvement were used. Fathers reported time spent with the focal child in the past month on a 5-point scale, with 1 = Every day or nearly every day, 2 = A few times a week, 3 = A few times in the last month, 4 = Only once or twice, and 5 = Not at all. For other domains of involvement, fathers reported the frequencies of engaging in respective activities using a 4-point scale, with 1 = Every day or almost every day, 2 = Several times a week, 3 = A few times in last month, and 4 = Never/Not at all. Due to the high skewness of the father involvement variables, the 11 items were recoded into binary variables. If fathers reported they never engaged in certain activities/behaviors, the responses were recoded as 0 = No. Otherwise, fathers’ responses were recoded as 1 = Yes, which indicated that fathers engaged in the activities/behaviors at least once in the past month.

#### 2.2.2. Child Social, Behavioral, and Cognitive Distal Outcomes

For detailed information on the construction of child outcome measures, please see [37], which included the description and results of factor analyses, tests of measurement equivalence, and tests of construct validity.

Child Social Competence. At the 30-month follow-up, mothers and fathers were independently interviewed about their children’s social competence. Parents evaluated nine items related to children’s interpersonal competence with peers, prosocial behavior, and friendship quality (e.g., “Resolves problems with other children on his or her own”) on a 3-point scale, ranging from 1 = Very True to 3 = Not True. The items were reverse coded and averaged so that higher scores represented higher child social competence. Both maternal reports (*α* = 0.84) and paternal reports (*α* = 0.85) showed good reliability. 

Child Internalizing and Externalizing Behavior Problems. At the 30-month follow-up, mothers and fathers were separately interviewed about their children’s internalizing and externalizing behavior problems. Parents were asked to indicate whether a list of behaviors accurately described their children’s behaviors by rating each behavior using a 3-point scale, ranging from 1 = Very True to 3 = Not True. If the focal child was 4 years old or older, the list of behaviors consisted of 12 items for internalizing behavior problems (e.g., “[Focal child] is unhappy, sad, or depressed”) and 13 items for externalizing behavior problems (e.g., “[Focal child] cheats or tells lies”). All the items were reverse coded so that higher scores indicated more behavior problems. The scales showed good reliability (internalizing: *α_M_* = 0.80 and *α_F_* = 0.80; externalizing: *α_M_* = 0.89 and *α_F_* = 0.87). If the focal child was under 4 years old, the list consisted of 8 items on internalizing behavior problems (e.g., “[Focal child] is too fearful or anxious”) and 14 items on externalizing behavior problems (e.g., “[Focal child] has difficulty concentrating and paying attention”). The reliability of the internalizing behavior problems scale was slightly low (*α_M_* = 0.61 and *α_F_* = 0.66), but the scale for externalizing behavior problems showed good reliability (*α_M_* = 0.81 and *α_F_* = 0.82). 

Verbal Ability/Cognitive Performance. At the 30-month follow-up, the Peabody Picture Vocabulary Test (PPVT) [38] and its Spanish-language counterpart, Test de Vocabulario en Imágenes Peabody (TVIP) [39], were used to measure children’s receptive vocabulary skills if focal children were aged 2 years to 4 years and 11 months. In these assessments, children were shown a series of cards with four pictures on each of them. In each trial, children were asked to choose one picture that best described the word spoken by the assessor. In the SHM program, some bilingual children were administered both the PPVT and TVIP. However, bilingual children received the TVIP only if they performed poorly at the beginning of the PPVT, which suggested that the TVIP was a more appropriate assessment for these children. Thus, we chose TVIP scores if children had scores on both tests. The standard scores were reported in the current study given that they were comparable across different studies [40]. 

#### 2.2.3. Covariates

To control for covariates, we created a set of variables based on prior literature. We controlled child age at the 30-month follow-up assessments. Child gender was entered as a binary variable in the analysis. We captured parental education using a dichotomous variable that identified whether both parents had high school diplomas or not. To assess poverty, we used the federal poverty level (FPL) and created two dummy variables (100–200% FPL, ≥200% FPL), with “below the FPL” as the reference group. 

### 2.3. Data Analysis

We first conducted descriptive statistics and correlation analysis across all study variables. To investigate potential heterogeneity in the patterns of father involvement, a latent class analysis (LCA) was conducted using the 11 items reflecting father involvement. LCA is an exploratory analytic method that is person-centered, allowing for the identification of hidden groups (or latent classes) based on multiple categorical observed variables, without requiring any distributional assumptions [41,42]. LCA provides probability estimates (posterior probability), which indicate how likely each individual belongs to each latent class [42,43]. In this paper, each latent class membership represents a group of fathers who share similar response patterns of involvement with their children. 

There are two common approaches to LCA, which are the one-step and three-step approaches, when including the covariates or distal outcomes. The one-step approach jointly estimates the latent class membership with the covariates or distal outcomes in one overall model. Thus, not only the class indicators, but also the covariates and the distal outcome variables, can drive the latent class membership [44,45]. On the other hand, the three-step approach employs a step-by-step method that identifies the latent classes in the first step, creates the most likely class membership in the second step, and estimates the association between the extracted latent class variable and the covariates or the distal outcomes in the last step. In this study, we used the three-step approach for LCA, which is advocated by many researchers [45,46,47,48,49]. More specifically, we employed the manual maximum likelihood (ML) three-step approach that has been shown to yield good performance in detecting latent classes [45]. 

In the first step, we analyzed a series of unconditional LCA models by increasing the number of latent classes from 2 classes to 6 classes. The unconditional LCA models represent the model with no covariates or distal outcomes but the 11 father involvement indicators only. We then compared the models using the Akaike information criterion (AIC) [50] and Bayesian information criterion (BIC) [51]. The AIC and BIC have slightly different formulas but are similar in that both penalize model complexity. The smaller AIC and BIC values represent a better-fitting model. Along with the AIC and BIC, entropy—which indicates the classification accuracy—was also used to decide the number of latent classes. The entropy value ranges between 0 and 1, with a value closer to 1 indicating a smaller model classification error [52]. The optimal number of latent classes was selected based on the AIC, BIC, and entropy, as well as the interpretability of the classes. Given the exploratory nature of LCA, we paid particular attention to the interpretability of the emerged latent classes (e.g., qualitatively distinct and meaningful classes). 

After identifying the number of latent classes based on the 11 indicators of father involvement, each individual was assigned to each latent class based on the posterior probability obtained in the second step. In the final step, we analyzed the conditional model to examine the mean differences in the seven measures of child social, behavioral, and cognitive distal outcomes across the enumerated latent classes while controlling for the covariate effects on the outcomes. Missing values were treated using full information maximum likelihood (FIML). In step 1, all cases (*N* = 2650) with at least one value across 11 indicators were used for analysis by using the maximum likelihood robust (MLR) estimation. Of the 2650 cases, 513 with at least one missing value in the covariates were excluded when conducting the third step. Mplus Version 8 [44] was used to conduct the three-step LCA (The Mplus code is presented in Supplemental Material Syntax S1). The study was approved by the Institutional Review Board of [Blinded for Review] (protocol ID: 2018B0532).

## 3. Results

### 3.1. Descriptive Statistics

Table 2 shows the descriptive statistics for all study variables, including father involvement indicators, covariates, and distal outcomes. As mentioned earlier, all father involvement indicators were recoded as dichotomous variables for the LCA. The original descriptive statistics for these items are presented in Supplemental Material Table S1.

### 3.2. Father Involvement Patterns

Table 3 shows the model fit indices, as well as the proportion of the emerged latent classes. The AIC continuously decreased as the number of classes increased, favoring more classes. The four-class model had the smallest BIC value, suggesting that the four-class model was the best-fitting model. The entropy was acceptable (>0.70) for models with four or more classes [53]. Based on the AIC, BIC, entropy, and interpretability of the classes, we selected the four-class model as the final model.

**Figure 1:**
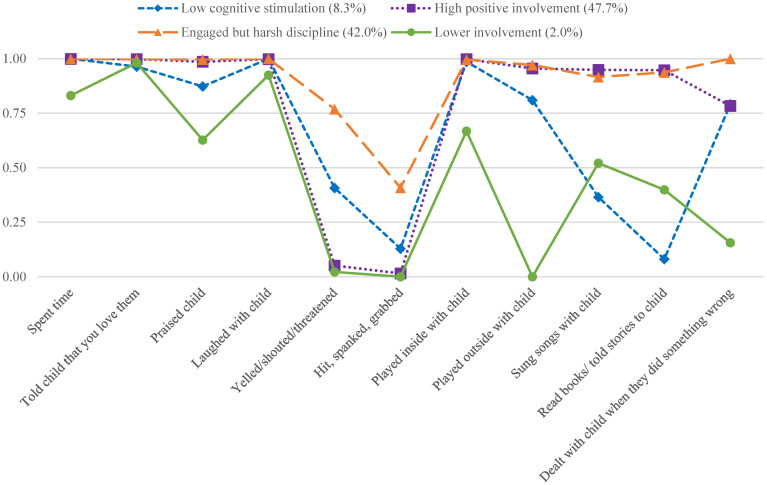
Item response probabilities for four father involvement latent classes.

Figure 1 shows the item response probability (IRP) on the 11 father involvement indicators for each latent class and class proportions. The *high positive involvement* class (47.48%) was the largest class and was characterized by high probabilities of positive involvement (e.g., time spent with child, warmth, engagement activities) and low probabilities of harsh emotional and physical discipline. The *engaged but harsh discipline* class (42.01%) represented the second-largest class and was also characterized by high probabilities of positive involvement (e.g., warmth, engagement activities), but also had the highest probabilities of harsh discipline out of all the classes. The *low cognitive stimulation* class (8.27%) was characterized by the lowest probabilities of paternal cognitive stimulation, but also moderately high probabilities of other aspects of positive involvement (e.g., time spent with child, warmth). The *lower involvement* class (2.04%) was characterized by overall low to moderate probabilities of all dimensions of father involvement examined in the study. 

### 3.3. Father Involvement Patterns and Child Social, Behavioral, and Cognitive Distal Outcomes

Next, we examined the extent to which different patterns of father involvement relate to children’s social competence, behavior problems, and verbal ability, while controlling for the effects of covariates on these outcomes (see Supplemental Material Table S2 for covariate effects). Table 4 presents the results of pair-wise mean comparisons for the seven child outcomes between the four latent classes. Children in the *low cognitive stimulation* class had significantly lower levels of socioemotional functioning class (distal means: father ratings = 2.16, mother ratings = 2.11) compared to those in the *high positive involvement* class (distal means: father ratings = 2.45, mother ratings = 2.37) or the *engaged but harsh discipline* class (distal means: father ratings = 2.41, mother ratings = 2.42). There were no significant mean differences in child socioemotional functioning between the *low cognitive stimulation* class and the *lower involvement* class.

For both internalizing and externalizing problems, children in the *low cognitive stimulation* class showed the highest levels of behavior problems among the four classes. The distal means of internalizing (father ratings = 1.53, mother ratings = 1.49) and externalizing (father ratings = 1.82, mother ratings = 1.79) behavior problems were significantly higher for children in the *low cognitive stimulation* class compared to children in the other three classes. The only exception was no significant difference in mother-reported externalizing behavior problems between the *low cognitive stimulation* class and the *lower involvement* class (mean difference = 0.35, *p* = 0.19). Additionally, children in the *engaged but harsh discipline* class showed significantly higher levels of father-reported internalizing problems than the *high positive involvement* and *lower involvement* classes, as well as significantly higher levels of father-reported externalizing problems than the *high positive involvement* class.

In terms of verbal/cognitive functioning, the mean PPVT score was significantly lower for children in the *low cognitive stimulation* class (*M* = 79.08) compared to those in the *high positive involvement* class (*M* = 85.03) and the *engaged but harsh discipline* class (*M* = 84.33). There were no other significant mean differences in PPVT scores across the latent classes.

## 4. Discussion

The primary aim of the study was to investigate whether and to what extent different patterns of father involvement are associated with various dimensions (e.g., social, behavioral, and cognitive) of child development among children in families with low income. Our findings contribute knowledge that can inform intervention efforts to foster healthy development among children in families with low income who are at heightened risk for negative developmental outcomes. Consistent with our hypotheses, we found heterogeneous patterns of father involvement. More specifically, we successfully identified four classes of father involvement that were qualitatively distinct from each other. These findings offer additional evidence and robust support for theoretical and empirical research that has suggested fathering is multidimensional [22,32,33,34]. Furthermore, the discovery of four distinctive patterns of father involvement provides empirical evidence for the heterogeneity in father involvement among low-income families, with some of the identified patterns consistent with prior research with families with low income [33,34].

### 4.1. Four Distinct Patterns of Involvement among Fathers

The largest proportion of the sample (47.48%) fell into the *high positive involvement* class in which fathers showed high levels of positive involvement (e.g., more time spent together, high paternal warmth and engagement) and low levels of harsh discipline. The finding that nearly half of the fathers in this sample exhibited the pattern of high, positive father involvement is especially important. Lower-income fathers, especially Black fathers, have often been depicted as invisible, absent, and uninvolved (e.g., “the myth of the missing Black father”) [54], yet our findings are consistent with other recent studies that challenge such stereotypes. For example, using a sample of fathers (in which close to half the sample was Black) from the Building Strong Families project, Lee et al. showed that fathers with a supportive parenting profile (i.e., highest levels of sensitivity, positive regard, cognitive stimulation, and the lowest levels of intrusiveness and detachment) made up the largest group out of the three distinct fathering groups they identified [34]. 

The second most prevalent pattern of father involvement was the *engaged but harsh discipline* class (42.01%) that was characterized by higher levels of involvement across the board, including greater use of harsh discipline and abusive behaviors. Relatively high probabilities of harsh discipline highlighted in this class are in line with prior research that identified a significant link between economic hardship and poor parenting, including child maltreatment [55]. Studies have suggested that economic hardship may introduce high parental stress, which may be associated with negative, harsh, and poor parenting behaviors [56]. Prior research with families with low income has found negative parenting profiles amongst fathers whereby they engage in moderate levels of sensitivity, cognitive stimulation, and positive regard along with high levels of intrusiveness and negative regard [33]. The *engaged but harsh discipline* class identified in our study is novel, however, given that no known fathering/parenting profile research has considered harsh discipline and abusive behaviors. Although such prior research has not used indicators of harsh discipline as in the case of the current study, the *engaged but harsh discipline* fathering class found in the current study seems to align with the negative fathering profile [33] in that they both share moderate to high levels of positive parenting behaviors within the context of high levels of poor parenting behaviors. 

The low cognitive stimulation class (8.27%) was distinguished by the lowest probabilities of paternal cognitive stimulation out of all classes. Fathers in this class showed particularly low levels of engagement (e.g., read books or tell stories to the child) in creating a cognitively stimulating, learning-rich home environment that can foster their children’s cognitive and language development. Fathers with low income and fathers of color may face multiple challenges (e.g., lack of time and resources, non-English speaking immigrants) that may serve as barriers to providing their children with cognitively stimulating environments and activities [57,58]. To the best of our knowledge, no similar fathering profile has been discovered in prior research with men with low income. This may be attributed to the fact that we used multiple indicators of cognitive stimulation (e.g., reading books, telling stories), whereas prior studies have only used a single observed measure of cognitive stimulation [33,34]. 

Lastly, the *lower involvement* class (2.04%), though the smallest in size, had distinct and meaningful differences from other classes. Fathers in this class showed generally lower levels of involvement in all dimensions of fathering, but especially with respect to the quantity of involvement (e.g., spending one or more hours a day with the child, playing inside with games or toys, taking the child for a walk or to play outside). This may be because fathers with low income tend to work more hours and have non-standard and/or changing work schedules [59]. The *lower involvement* class seems to be consistent with the detached parenting profile found amongst fathers with low income [33], whereby fathers exhibit generally low levels of engagement in both positive and negative parenting behaviors. Importantly, considering its size, this class should be replicated and validated with other samples in future research.

### 4.2. Father Involvement Patterns and Children’s Developmental Outcomes

In terms of the relations between father involvement patterns and child development, one of the most notable findings was the important role played by paternal cognitive stimulation in child development among families with low income. Children in the *low cognitive stimulation* class struggled across the socioemotional, behavioral, and cognitive domains of development, showing higher levels of father- and mother-reported behavior problems and lower levels of socioemotional and cognitive functioning compared to the other three groups. These findings are consistent with the broader literature that report the positive association between fathers’ cognitive stimulation (e.g., stimulating parenting, reading books to children, fathers’ home literacy involvement) and children’s healthy development during early childhood [21,60,61,62]. While much of the prior work documented the impact of cognitive stimulation on children’s cognitive development, such as verbal ability, language outcomes, and academic skills [3,61,63], our findings suggest that the positive influence of paternal cognitive stimulation expands beyond cognitive development into other domains of child development, such as socioemotional and behavioral functioning. 

Another primary finding was that children in the *engaged but harsh discipline* class had significantly higher levels of father-reported internalizing problems (than the *high positive involvement* and *lower involvement* classes) and externalizing problems (than the *high positive involvement* class). This finding implies that the high level of involvement in other aspects of fathering (e.g., warmth, cognitive stimulation, time spent together) did not buffer the negative impact of harsh discipline and abusive behaviors on children’s behavioral outcomes. Our findings are largely consistent with previous studies that found higher levels of internalizing and externalizing symptoms among children who have experienced harsh discipline, including physical punishment and emotional/verbal abuse [64,65]. The family stress model and prior empirical studies suggest that economic hardship and financial pressure in fathers/parents with low income may be related to disrupted parenting practices through elevated levels of psychological distress and interparental conflict [66]. Drawing from social learning theory [67], disruptive parenting (e.g., harsh discipline), in turn, could be associated with negative child adjustment as children exposed to violent acts may observe and model aggressive behavior. 

It should also be noted that the *engaged but harsh discipline* class was related to greater behavior problems reported by fathers, but not mothers. This may be explained by the reciprocal associations between paternal harsh discipline and children’s behavior problems over time [68]. That is, fathers who see their children as having behavior problems (i.e., internalizing and externalizing symptoms) may be more likely to use harsh physical and verbal discipline to manage or correct their children’s problem behaviors, and the use of harsh discipline may further exacerbate children’s problem behaviors (e.g., children become more aggressive and antisocial). However, more research is needed to disentangle the complex associations between paternal harsh discipline and child behavior problems and understand the discrepancies in findings between different informants (i.e., fathers vs. mothers).

### 4.3. Limitations

This study has several limitations. First, because the study sample consisted of married couples with low income and residential fathers who participated in the SHM intervention, the findings of the study may not be generalizable to a broader population. In particular, SHM participants volunteered to receive healthy relationship and marriage strengthening education and services by participating in the project. Second, the study relied solely on fathers’ reports to assess father involvement. The use of multiple informants (e.g., both mothers and fathers) may provide a more nuanced and fuller picture of father involvement. Relatedly, we were unable to include maternal involvement items in the analytic models due to the heavy skewness of the items (i.e., a lack of variability). Another measurement-related limitation is that some of the measures used in this study, including father involvement items, have not been standardized or validated in prior studies or with families with low income. The results of the study should be interpreted with caution and considered somewhat preliminary, in light of these measurement limitations. Third, the size of the *lower involvement* class was small (2% of the sample). Although the *lower involvement* class represented a distinct pattern of father involvement observed among fathers with low income, this class should be replicated and validated with other samples of fathers with low income in future research to establish greater reliability. Fourth, there were potentially important factors, such as the quality of father–mother relationships, child temperament, and race/ethnicity, that were not accounted for in the current study, either due to lack of data or given the complexity of the analysis employed in the study. Future research should explore how various biological and environmental factors might be related to patterns of father involvement and child development in families with low income. Finally, any causal inferences cannot be drawn from this study due to the nature of the study design.

### 4.4. Implications for Policy and Practice

The current study offers several important implications for policy and practice. Our results highlight the significance of positive father involvement in healthy child development. At the policy level, increased funding and resources should be allocated to support programs and initiatives (e.g., responsible fatherhood programs) that encourage and facilitate positive father involvement in the lives of children among families with low income. At the practice level, more effort is needed to actively engage fathers in parenting interventions, services, and programs. Our finding that fathers’ cognitive stimulation is a key promotive factor for children’s healthy social, behavioral, and cognitive development points to the need for fatherhood programming to include components that focus on enhancing fathers’ involvement in activities that are cognitively stimulating for their children. For example, practitioners working with fathers with low income could help fathers create a language-rich and cognitively stimulating home environment and build skills to interact with their children in ways that promote language and cognitive development (e.g., reading books the child, using educational materials, telling stories, singing songs, etc.) [69]. Further, considering that paternal harsh discipline was a salient risk factor for behavior problems in children, efforts to engage fathers in programs that focus on positive parenting and maltreatment prevention are needed. 

## 5. Conclusions

The findings of the study contribute to a body of emerging research examining patterns of father involvement among families with low income. The identification of four father involvement patterns (i.e., *high positive involvement*; *engaged but harsh discipline*; *low cognitive stimulation*; *lower involvement*) and their unique associations with child development provide meaningful information that can be incorporated into interventions for young children in socioeconomically disadvantaged families. Given that our findings highlight the pivotal role of positive father involvement, such as paternal cognitive stimulation, on healthy child development, researchers and clinicians developing interventions for positive child development should consider actively engaging fathers in intervention programs and services. Finally, future research should explore potential differences and diversity in patterns of father involvement across different racial/ethnic groups and different developmental stages of children. 

## Figures and Tables

**Table 1:** Demographic Characteristics (*N* = 2650).

			% or *M* (SD)
Father			
Age			29.35 (5.84)
Race and ethnicity	White		45.60
	African American		19.23
	Asian		2.40
	American Indian/Alaska Native		4.16
	Pacific Islander		1.47
	Others		27.14
	Hispanic		40.48
Education	At least a high school diploma		80.10
Residential status	15-month follow-up	Lived with child at least half of the time	97.25
	30-month follow-up		91.97
Mother			
Age			27.40 (5.26)
Race and ethnicity	White		48.81
	African American		14.70
	Asian		3.20
	Native American		4.17
	Pacific Islander		1.58
	Others		27.54
	Hispanic		40.65
Education	At least a high school diploma		81.50
Couple			
Marital Status	12-month follow-up	Married	85.29
		In a committed relationship	10.10
		Divorced	0.95
		Separated	3.67
	30-month follow-up	Married	79.44
		In a committed relationship	9.56
		Divorced	3.07
		Separated	7.93
Household			
Income	Below the federal poverty level (FPL)		38.12
	Between 100% and 200% of FPL		41.99
	Above 200% FPL		19.89
Focal Child			
Gender	Boy		51.79
	Girl		48.21

**Table 2:** Descriptive statistics for the indicators, covariates, and distal outcomes (*N* = 2650).

Dimensions of Father Involvement at the 12-Month Follow-Up		%
Time spent	Spend one or more hours a day with the child	99.58
Warmth	Told (focal child) that you love (him/her)?	99.47
	Praised (focal child) or told him/her that you appreciated something that he/she did?	97.43
	Laughed with (focal child)?	99.77
Harsh discipline	Yelled, shouted, screamed at, or threatened (focal child) because you were mad at him/her?	38.12
	Hit, spanked, grabbed, or used physical punishment with (focal child)?	18.91
Engagement	Played inside with games or toys	98.98
	Taken the child for a walk or to play outside	93.00
	Sung songs or nursery rhymes with the child	87.78
	Read books or told stories to the child	86.01
	Dealt with the children when he/she did something wrong	86.26
Covariates at Baseline		% or *M* (SD)
Child age (at the 30-month follow up)		3.66 (1.32)
Child sex (girl)		48.2
Couple education (both graduated from high school)		56.6
Poverty		
100% of federal poverty level or under		38.1
Between 100% and 200% of federal poverty level		42.0
200% of federal poverty level or above		19.9
Distal Child Development Outcomes at the 30-Month Follow-Up		*M* (SD)
Social emotional functioning assessed by father		2.57 (0.37)
Social emotional functioning assessed by mother		2.56 (0.37)
Internalizing behavior problem assessed by father		1.21 (0.25)
Internalizing behavior problem assessed by mother		1.19 (0.25)
Externalizing behavior problem assessed by father		1.34 (0.30)
Externalizing behavior problem assessed by mother		1.36 (0.32)
Cognitive functioning (verbal ability) assessed by interviewer		97.29 (15.97)

**Table 3:** Fit indices for unconditional latent class models.

	2-Class	3-Class	4-Class	5-Class	6-Class
Log-Likelihood	−7128.50	−6860.18	−6792.45	−6757.93	−6739.75
Number of parameters	23	35	47	59	71
AIC	14,303.00	13,790.36	13,678.90	13,633.85	13,621.51
BIC	14,438.30	13,996.24	13,955.37	13,980.91	14,039.15
Entropy	0.54	0.68	0.75	0.75	0.78
Proportion of class 1	49.97%	46.47%	8.27%	45.31%	5.34%
Proportion of class 2	50.03%	44.13%	47.48%	7.23%	4.48%
Proportion of class 3		9.41%	42.01%	40.79%	41.26%
Proportion of class 4			2.04%	0.81%	45.79%
Proportion of class 5				5.86%	2.68%
Proportion of class 6					0.45%

**Table 4:** Distal mean differences between four latent classes.

Child Distal Outcome	Class	Distal Mean	Low Cognitive Stimulation	High Positive Involvement	Engaged but Harsh Discipline
Socioemotional functioning _father ratings	Low cognitive stimulation	2.16			
	High positive involvement	2.45	−0.29 ***		
	Engaged but harsh discipline	2.41	−0.25 ***	0.04	
	Lower involvement	2.31	−0.15	0.14	0.10
Socioemotional functioning _mother ratings	Low cognitive stimulation	2.11			
	High positive involvement	2.37	−0.26 ***		
	Engaged but harsh discipline	2.42	−0.31 ***	−0.05	
	Lower involvement	2.29	−0.18	0.08	0.13
Internalizing problems _father ratings	Low cognitive stimulation	1.53			
	High positive involvement	1.11	0.42 ***		
	Engaged but harsh discipline	1.23	0.30 ***	−0.12 ***	
	Lower involvement	1.13	0.40 ***	−0.02	0.10 ***
Internalizing problems _mother ratings	Low cognitive stimulation	1.49			
	High positive involvement	1.14	0.35 ***		
	Engaged but harsh discipline	1.11	0.38 ***	0.03	
	Lower involvement	1.15	0.34 ***	−0.01	−0.04
Externalizing problems _father ratings	Low cognitive stimulation	1.82			
	High positive involvement	1.33	0.49 ***		
	Engaged but harsh discipline	1.47	0.35 ***	−0.14 **	
	Lower involvement	1.39	0.43 ***	−0.06	0.08
Externalizing problems _mother ratings	Low cognitive stimulation	1.79			
	High positive involvement	1.39	0.40 ***		
	Engaged but harsh discipline	1.38	0.41 ***	0.01	
	Lower involvement	1.44	0.35	−0.05	−0.06
Child cognitive functioning _interviewer ratings	Low cognitive stimulation	79.08			
	High positive involvement	85.03	−5.95 **		
	Engaged but harsh discipline	84.33	−5.25 **	0.70	
	Lower involvement	78.37	0.71	6.66	5.96

Note. ***: *p* < 0.001; **: *p* < 0.01

## Data Availability

Restrictions apply to the availability of these data. Data were obtained from the Inter-university Consortium for Political and Social Research.

## Supplementary Materials

The following are available online at https://www.mdpi.com/article/10.3390/children8121164/s1, Table S1: Descriptive statistics for the indicators, covariates, and distal outcomes, Table S2: Effects of the covariates on the distal outcomes, Syntax S1: Mplus syntax.
