# Supplementary material for: Patterns of Father Involvement and Child Development among Families with Low Income
Source: Children (Basel). 2021 Dec 9;8(12):1164. doi: 10.3390/children8121164 (PMC8699948; doi:10.3390/children8121164)
Supplement: Supplementary file 1 [file children-08-01164-s001.zip › children-1474455-supplementary.pdf]

Supplemental Material A.

Table S1

Demographic Characteristics (N = 2,650).

			%	Mean	SD
**Father**					
Age				29.35	5.84
Race and ethnicity	White		45.60		
	African American		19.23		
	Asian		2.40		
	Native American		4.16		
	Pacific Islander		1.47		
	Others		27.14		
	Hispanic		40.48		
Education	At least a high school diploma		80.10		
Residential status	15 months follow-up	Lived with children at least half of the time	97.25		
	30 months follow-up		91.97		
**Mother**					
Age				27.40	5.26
Race and ethnicity	White		48.81		
	African American		14.70		
	Asian		3.20		
	Native American		4.17		
	Pacific Islander		1.58		
	Others		27.54		
	Hispanic		40.65		
Education	At least a high school diploma		81.50		
**Couple**					
Marital Status	12-month follow-up	Married	85.29		
		In a committed relationship or romantically involved	10.10		
		Divorced	0.95		
		Separated	3.67		
	30-month follow-up	Married	79.44		
		In a committed relationship or romantically involved	9.56		
		Divorced	3.07		
		Separated	7.93		
**Household**					
Income	Below the Federal Poverty Level (FPL)		38.12		
	Between 100% and 200% of FPL		41.99		
	Above 200% FPL		19.89		
**Focal Child**					
Gender	Boy		51.79		
	Girl		48.21		

Supplemental Material B.

Table S2

Descriptive Statistics for the Indicators, Covariates, and Distal Outcomes (N = 2,650).

Father involvement		M (SD)	Skewness	Kurtosis	%
Time spent	Spend one or more hours a day with the child	4.84 (0.51)	-4.13	20.54	99.58
Paternal warmth	Told (focal child) that you love (him/her)?	3.92 (0.36)	-5.37	31.39	99.47
	Praised (focal child) or told him/her that you appreciated something that he/she did?	3.78 (0.61)	-3.17	9.93	97.43
	Laughed with (focal child)?	3.93 (0.32)	-5.21	30.59	99.77
Harsh discipline	Yelled, shouted, screamed at, or threatened (focal child) because you were mad at him/her?	1.59 (0.88)	1.37	0.88	38.12
	Hit, spanked, grabbed or used physical punishment with (focal child)?	1.24 (0.55)	2.50	6.39	18.91
Father's engagement	Played inside with games or toys	3.76 (0.54)	-2.61	7.32	98.98
	Taken the child for a walk or to play outside	3.01 (0.91)	-0.58	-0.53	93.00
	Sung songs or nursery rhymes with the child	3.10 (1.04)	-0.84	-0.57	87.78
	Read books or told stories to the child	2.93 (1.06)	-0.55	-0.98	86.01
	Dealt with the children when he/she did something wrong	3.21 (1.09)	-1.06	-0.35	86.26

Supplemental Material C.

Table S3

Effects of the Covariates on the Distal Outcomes.

Distal outcome	Covariates	Estimate	S.E.	Estimate/ S.E.	*p-value*
Social emotional functioning assessed by father	Child age	0.05***	0.01	8.13	0.000
	Child sex	0.02	0.02	1.06	0.288
	Couple education	-0.02	0.02	-1.03	0.305
	Poverty level-2	-0.00	0.02	-0.10	0.920
	Poverty level-3	-0.03	0.02	-1.27	0.205
Social emotional Functioning assessed by mother	Child age	0.05***	0.01	7.90	0.000
	Child sex	0.03*	0.02	2.21	0.027
	Couple education	-0.02	0.02	-1.30	0.195
	Poverty level-2	0.03	0.02	1.80	0.072
	Poverty level-3	0.04	0.02	1.94	0.052
Child internalizing behavior problem assessed by father	Child age	-0.00	0.00	-1.00	0.319
	Child sex	0.00	0.01	0.31	0.756
	Couple education	0.00	0.01	0.21	0.831
	Poverty level-2	-0.01	0.01	-1.21	0.228
	Poverty level-3	-0.02	0.01	-1.89	0.059
Child internalizing behavior problem assessed by mother	Child age	0.01*	0.00	2.15	0.032
	Child sex	0.00	0.01	0.44	0.662
	Couple education	-0.02*	0.01	-2.24	0.025
	Poverty level-2	-0.02*	0.01	-1.99	0.047
	Poverty level-3	-0.02	0.01	-1.75	0.080
Child externalizing behavior problem assessed by father	Child age	-0.04***	0.00	-7.94	0.000
	Child sex	0.00	0.01	0.20	0.844
	Couple education	0.02	0.01	1.66	0.096
	Poverty level-2	-0.02	0.01	-1.25	0.212
	Poverty level-3	-0.01	0.02	-0.60	0.551
Child externalizing behavior problem assessed by mother	Child age	-0.02***	0.01	-4.23	0.000
	Child sex	-0.00	0.01	-0.21	0.833
	Couple education	-0.02	0.01	-1.43	0.154
	Poverty level-2	-0.01	0.01	-0.36	0.722
	Poverty level-3	0.01	0.02	0.28	0.779
PPVT	Child age	2.30**	0.69	3.36	0.001
	Child sex	2.79**	0.98	2.85	0.004
	Couple education	2.86**	1.10	2.61	0.009
	Poverty level-2	2.81*	1.12	2.52	0.012
	Poverty level-3	7.35***	1.50	4.89	0.000

Note. *: $p < .05$; **: $p < .01$; ***: $p < .001$.

Supplemental Material D.

Mplus syntax for the first step

```
DATA: FILE IS LCA_ step1.csv;

VARIABLE: NAMES ARE id spentime warmth1 warmth2 warmth3 emodisc phydisc
          playaver playin playout commaver song book dealwith
          !father involvement indicators
          chilagey chilgen edubhigh poverty2 poverty3
          !covariates
          emofun_h emofun_w inbfun_h inbfun_w exbfun_h exbfun_w ppvt;
          !distal outcomes
          USEVARIABLES ARE spentime warmth1 warmth2 warmth3 emodisc phydisc
          playin playout song book dealwith;
          CATEGORICAL = spentime warmth1 warmth2 warmth3 emodisc phydisc
          playin playout song book dealwith;
          AUXILIARY = chilagey chilgen edubhigh poverty2 poverty3
          !covariates
          emofun_h emofun_w inbfun_h inbfun_w exbfun_h exbfun_w ppvt;
          !distal outcomes
          MISSING ARE ALL (-999);
          CLASSES = c(4);

ANALYSIS: TYPE=MIXTURE;
          STARTS = 500 50;
          STITERATIONS = 20;

OUTPUT: TECH11 TECH14 SVALUES;

PLOT: TYPE = PLOT3;
      SERIES = spentime warmth1 warmth2 warmth3 emodisc phydisc
      playin playout song book dealwith(*);

SAVEDATA: FILE IS Father_involvement_LCA_from_step1.txt;
          SAVE IS cprob;
          MISSFLAG = 999;
```

Mplus syntax for the third step

```
DATA: FILE IS Father_involvement_LCA_from_step1.txt;

VARIABLE: NAMES ARE spentime warmth1 warmth2 warmth3 emodisc phydisc
                playin playout song book dealwith
                !father involvement
                chilagey chilgen edubhigh poverty2 poverty3
                !control variables
                emofun_h emofun_w inbfun_h inbfun_w exbfun_h exbfun_w ppvt
                !distal outcomes
                cprob1 cprob2 cprob3 cprob4 n;
                !cprobabilities and modal class
                 USEVARIABLES ARE chilagey chilgen edubhigh poverty2 poverty3
                !control variables
                emofun_h emofun_w inbfun_h inbfun_w exbfun_h exbfun_w ppvt
                !distal outcomes
                n;
                !modal class
                NOMINAL = n;
                MISSING ARE ALL (999);
                CLASSES = c(4);

ANALYSIS: TYPE=MIXTURE;
                 STARTS = 500 50;
                 STITERATIONS = 20;

MODEL:
%OVERALL%
emofun_h ON chilagey chilgen edubhigh poverty2 poverty3;
emofun_w ON chilagey chilgen edubhigh poverty2 poverty3;
inbfun_h ON chilagey chilgen edubhigh poverty2 poverty3;
inbfun_w ON chilagey chilgen edubhigh poverty2 poverty3;
exbfun_h ON chilagey chilgen edubhigh poverty2 poverty3;
exbfun_w ON chilagey chilgen edubhigh poverty2 poverty3;
ppvt ON chilagey chilgen edubhigh poverty2 poverty3;

%C#1%
[n#1@3.351]; !logit from the result of step 1
[n#2@1.923]; !logit from the result of step 1
[n#3@1.627]; !logit from the result of step 1
[emofun_h] (efh1);
[emofun_w] (efw1);
[inbfun_h] (ibh1);
[inbfun_w] (ibw1);
[exbfun_h] (ebh1);
```

```
[exbfun_w] (ebw1);
[ppvt] (ppvt1);
emofun_h;
emofun_w;
inbfun_h;
inbfun_w;
exbfun_h;
exbfun_w;
ppvt;

%C#2%
[n#1@1.125]; !logit from the result of step 1
[n#2@6.123]; !logit from the result of step 1
[n#3@3.251]; !logit from the result of step 1
[emofun_h] (efh2);
[emofun_w] (efw2);
[inbfun_h] (ibh2);
[inbfun_w] (ibw2);
[exbfun_h] (ebh2);
[exbfun_w] (ebw2);
[ppvt] (ppvt2);
emofun_h;
emofun_w;
inbfun_h;
inbfun_w;
exbfun_h;
exbfun_w;
ppvt;

%C#3%
[n#1@4.899]; !logit from the result of step 1
[n#2@7.612]; !logit from the result of step 1
[n#3@9.445]; !logit from the result of step 1
[emofun_h] (efh3);
[emofun_w] (efw3);
[inbfun_h] (ibh3);
[inbfun_w] (ibw3);
[exbfun_h] (ebh3);
[exbfun_w] (ebw3);
[ppvt] (ppvt3);
emofun_h;
emofun_w;
inbfun_h;
inbfun_w;
exbfun_h;
exbfun_w;
```

```
ppvt;

%C#4%
[n#1@-2.670]; !logit from the result of step 1
[n#2@-2.234]; !logit from the result of step 1
[n#3@-8.082]; !logit from the result of step 1
[emofun_h] (efh4);
[emofun_w] (efw4);
[inbfun_h] (ibh4);
[inbfun_w] (ibw4);
[exbfun_h] (ebh4);
[exbfun_w] (ebw4);
[ppvt] (ppvt4);
emofun_h;
emofun_w;
inbfun_h;
inbfun_w;
exbfun_h;
exbfun_w;
ppvt;

MODEL CONSTRAINT:
New(efh1v2 efh1v3 efh1v4 efh2v3 efh2v4 efh3v4
efw1v2 efw1v3 efw1v4 efw2v3 efw2v4 efw3v4
ibh1v2 ibh1v3 ibh1v4 ibh2v3 ibh2v4 ibh3v4
ibw1v2 ibw1v3 ibw1v4 ibw2v3 ibw2v4 ibw3v4
ebh1v2 ebh1v3 ebh1v4 ebh2v3 ebh2v4 ebh3v4
ebw1v2 ebw1v3 ebw1v4 ebw2v3 ebw2v4 ebw3v4
ppvt1v2 ppvt1v3 ppvt1v4 ppvt2v3 ppvt2v4 ppvt3v4);

efh1v2 = efh1 - efh2;
efh1v3 = efh1 - efh3;
efh1v4 = efh1 - efh4;
efh2v3 = efh2 - efh3;
efh2v4 = efh2 - efh4;
efh3v4 = efh3 - efh4;

efw1v2 = efw1 - efw2;
efw1v3 = efw1 - efw3;
efw1v4 = efw1 - efw4;
efw2v3 = efw2 - efw3;
efw2v4 = efw2 - efw4;
efw3v4 = efw3 - efw4;

ibh1v2 = ibh1 - ibh2;
ibh1v3 = ibh1 - ibh3;
```

ibh1v4 = ibh1 - ibh4;
ibh2v3 = ibh2 - ibh3;
ibh2v4 = ibh2 - ibh4;
ibh3v4 = ibh3 - ibh4;

ibw1v2 = ibw1 - ibw2;
ibw1v3 = ibw1 - ibw3;
ibw1v4 = ibw1 - ibw4;
ibw2v3 = ibw2 - ibw3;
ibw2v4 = ibw2 - ibw4;
ibw3v4 = ibw3 - ibw4;

ebh1v2 = ebh1 - ebh2;
ebh1v3 = ebh1 - ebh3;
ebh1v4 = ebh1 - ebh4;
ebh2v3 = ebh2 - ebh3;
ebh2v4 = ebh2 - ebh4;
ebh3v4 = ebh3 - ebh4;

ebw1v2 = ebw1 - ebw2;
ebw1v3 = ebw1 - ebw3;
ebw1v4 = ebw1 - ebw4;
ebw2v3 = ebw2 - ebw3;
ebw2v4 = ebw2 - ebw4;
ebw3v4 = ebw3 - ebw4;

ppvt1v2 = ppvt1 - ppvt2;
ppvt1v3 = ppvt1 - ppvt3;
ppvt1v4 = ppvt1 - ppvt4;
ppvt2v3 = ppvt2 - ppvt3;
ppvt2v4 = ppvt2 - ppvt4;
ppvt3v4 = ppvt3 - ppvt4;
